# Advancing smart communities with a deep learning framework for sustainable resource management

**DOI:** 10.1371/journal.pone.0329492

**Published:** 2025-08-07

**Authors:** Yongyan Zhao

**Affiliations:** School of Humanities and Law, Harbin University, Harbin, Heilongjiang, China; The Hong Kong Polytechnic University, CHINA

## Abstract

**Background:**

The rapid development of urban systems and rising requirements for sustainable development lift resource management issues in smart communities. A fundamental problem for contemporary communities involves effectively using energy and water resources and waste management systems under environmental limitations. Artificial intelligence (AI) techniques at an advanced level deliver new methods that optimize resource management systems.

**Objective:**

The research builds and examines a deep-learning framework that optimizes the management of smart community resources. The framework leverages long short-term memory (LSTM) networks for temporal data, convolutional neural networks (CNNs) for spatial analysis, and autoencoders for anomaly detection. The system focuses on two main objectives, which include better forecasting precision, optimum resource distribution, and efficient detection of operational problems.

**Methods:**

Research validation employed data from the Amsterdam Open Data Platform and Singapore Government Open Data Portal joined by crowdsourced platforms FixMyStreet and OneService. The preprocessing phase involved three stages, i.e., cleaning and normalization and feature engineering steps, before model training and testing phases. Predictive models received assessment based on Mean Absolute Error (MAE), Root Mean Squared Error (RMSE), and R². A comparison with traditional methods revealed the proposed approach delivered superior performance results.

**Results:**

The deep learning framework demonstrated superior performance, achieving an average reduction of 18.7% in resource consumption and a 16.2% reduction in operational costs. The models outperformed baseline methods, with LSTMs achieving an MAE of 1.8 for water demand prediction and autoencoders detecting anomalies with an F1-score of 95.5%.

**Conclusion:**

Due to its effective capabilities, the proposed framework solves challenges in resource management for smart communities while showing the potential of AI-driven solutions for sustainable urban development. Research results demonstrate that integrating sophisticated deep-learning methods yields more significant potential for optimizing resource utilization while improving operational effectiveness.

## Introduction

The rapid increase of urban populations throughout the 21st century indicates that urban areas will hold a 68% occupancy rate by 2050 [1]. An exponential growth of urban populations increases pressure on natural resources, so we need more sustainable management practices now than ever. Smart communities represent the foundational approach to handling environmental challenges. Smart communities implement advanced technologies, including the Internet of Things (IoT), together with artificial intelligence (AI) and big data analytics to build efficient, sustainable cities [2]. Smart communities gain essential support from deep learning technology, which shares roots with AI in providing machines with an advanced data analysis and processing method. Pattern detection happens automatically with prediction and the fundamental design aspect of complex neural networks in deep learning models, which needs minimal human supervision [3]. Deep learning brings highly effective analytical tools to address complex resource management issues encompassing electricity grid merging, water optimization, and waste path optimization [4]. Research motivation arises because Smart community developers must integrate their initiatives with the United Nations Sustainable Development Goals (SDG) to achieve clean energy, sustainable cities, and responsible consumption targets [5]. Smart communities currently use single-purpose applications, consisting of traffic control systems and bright lighting, as their primary methods for resource management. Current real-world large scalable applications demonstrate the sparse application of deep learning methods to achieve full domain integration in resource management [6]. Recent developments in hardware resources alongside cloud computing abilities have transformed deep learning model deployment accessibility even for constrained resource systems [7]. This research establishes a complete framework to unite deep learning capabilities within smart community solutions while addressing current research deficiencies. It aims to show these technologies’ abilities to effectively manage sustainable resources while strengthening urban resilience and boosting community member quality of life. The research adds to smart urban development literature by resolving identified gaps and presenting novel solutions to deal with present environmental and social urban issues.

Modern urban planning incorporates smart communities as vital foundation elements that unite innovative technologies with social, economic, and environmental systems to boost resident life quality. Sustainable urban areas exist to confront rapid growth difficulties regarding limited resources, environmental damage, and infrastructure space problems, which support economic development and social harmony [8]. Real-time data serves as a fundamental requirement for smart communities to optimize the functions of their urban system. The dynamic balance of energy supply meets demand through smart energy grids, and smart water management systems perform effective water conservation and allocation at the same time [9]. Smart transportation networks enhance traffic optimization along with lower carbon emissions and successfully address the sustainability goals of urban development [10]. Within smart communities, the focus remains on giving residents better ways to take part in decisions and interact with services efficiently. Real-time data from digital platforms and Internet of Things devices enables users to monitor their energy use, water patterns, and waste output, therefore developing a feeling of collective responsibility within the group [11]. Smart communities that use sustainability principles alongside modern technology achieve compliance with international frameworks, especially through the UN’s Sustainable Development Goals and their targets toward sustainable cities and communities [12]. The smart communities approach serves the evolving urban development market through its technologically enhanced system for fostering sustainable inclusivity and resilience.

The emergence of deep learning tools has become essential for managing complex resource problems because they detect hidden patterns contained within extensive datasets that traditional processes commonly fail to observe. Deep learning models support multi-dimensional neural networks that analyze unstructured datasets effectively, which makes them optimal for urban system optimization [13]. Implementing deep learning has proven crucial to multiple domains within resource management. Deep learning models use prediction algorithms to detect future electricity demand patterns for optimizing utility energy management and grid system control [14]. According to research, water resource management systems use neural networks to predict consumption patterns and identify distribution network leaks [15]. Through real-time identification processes, deep learning-based computer vision systems facilitate precise waste management practices by sorting waste materials [16]. Deep learning technology produces predictive results essential for companies’ strategic planning decisions. By using deep learning as a power source for climate models, we can make accurate predictions about extreme weather, which helps communities develop plans for preparation and resource protection [17]. Deep learning models now find deployment in limited-resource settings through edge computing with cloud platforms, which expands their reach into secluded areas [18]. Deep learning applications face implementation obstacles despite showing promising potential in resource management usage. Delectable data security problems, hefty processor requirements, and significant training datasets need solution methods for fruitful deployment. Deep learning develops into an indispensable component of smart community frameworks because of its transformative capability to optimize resource usage while lowering environmental impact and building system efficiency.

The primary objective of this research is to explore and develop a comprehensive framework for integrating deep learning into smart community systems to achieve sustainable resource management. This study aims to:

- To analyze the potential applications of deep learning in optimizing resource usage, such as energy efficiency, water management, and waste reduction.
- To propose an adaptable deep learning-based model for real-time resource allocation and monitoring decision-making.
- To assess the performance of the proposed framework in improving resource efficiency, reducing waste, and promoting sustainability.
- To ensure the proposed solution can be scaled across diverse urban settings and resource-constrained environments.

These objectives aim to contribute to the advancement of smart communities by providing innovative, data-driven approaches to sustainable urban development.

A comprehensive framework uses deep learning approaches to improve smart community resource administration operations. The proposed modeling approach combines IoT devices with public datasets and crowdsourced entries to execute exact resource demand predictions while detecting anomalies and optimizing energy and water waste operations. According to this research study, advanced neural networks, including autoencoders and LSTMs, successfully boost operational efficiency and prediction accuracy.

The work includes a systematic organization that details the proposed framework and its effects on resource management practices. Subsequent literature review sections focus on research gaps within existing approaches. The methodology section specifies deep learning methods, data sources, and the proposed computational model. The model’s comprehensive performance analysis emerges through a discussion of results while assessing its resource optimization capabilities and traditional approaches. The conclusion summarizes significant research contributions while suggesting potential pathways to improve sustainable resource management techniques within smart communities.

## Related work

The evolution of smart communities as a multidisciplinary research field has gained momentum since the late 1990s, addressing the challenges posed by rapid urbanization, resource shortages, and climate change. A smart community integrates IoT, artificial intelligence (AI), and big data analytics to develop sustainable, technology-driven infrastructures that improve quality of life. While deep learning has shown increasing success in smart community applications, significant research gaps remain in resource management implementations, limiting the scalability and inclusivity of these solutions. [19]. The current research lacks integration frameworks to unify single-application systems, which would create an environment for comprehensive resource management in smart communities. Community development toward smart cities demands multiple domain management and interconnected infrastructure optimization to create coordinated operations between separate systems. Multiple trial implementations and case studies show advanced community technologies produce effective outcomes, yet widespread deployment faces significant scaling barriers. Today’s Research does not capture the multifold implementation challenges of these technologies when deployed on extensive scales throughout entire cities and regions, specifically in resource-limited settings [20]. Most prior research applies standalone deep learning models such as LSTMs for time-series forecasting, CNNs for spatial analysis, or traditional machine learning algorithms for anomaly detection. However, these approaches fail to capture the interdependencies between resource domains and lack adaptability for scaling across large urban environments. Several experimental studies and pilot projects have demonstrated promising results, yet scalability challenges persist, particularly in resource-constrained regions where computational costs and infrastructure limitations hinder widespread adoption [21]. Their limited applicability prevents widespread deployment because models cannot adapt to various environmental conditions. Researchers must create flexible universal frameworks to implement across urban and rural locations. Smart communities incorporating IoT devices and data-driven platforms encounter widespread security and privacy risks with broad deployment. Research activity today mainly deals with technological methods of information acquisition and processing. The research falls short of addressing ethical matters relating to citizen data privacy and system security design while omitting necessary policy structures needed for protection [22]. Studies in smart community technology focus on user engagement as a complementary aspect rather than establishing engagement as a fundamental part [23]. Current research fragments the identification of techniques that empower citizens to actively participate in resource management through deep learning-based systems enabled by artificial intelligence. The journal publications pay scant attention to the environmental impact of sophisticated technological solutions regardless of the deep learning model of electricity requirements. The study on how these technologies shape community diversity and inclusivity is insufficiently researched [24]. In the future, research needs to examine both the technical specifications and the full environmental and social ramifications of smart community systems. Industrial innovations move faster than the creation of adequate policies, which allows the coherent implementation of advanced technologies. Research fails to study and test essential legal frameworks enabling deep learning platforms and other advanced smart community technology platforms within existing frameworks [25]. The reforms will require extensive teamwork between experts from technical fields, social specialists, and policy professionals. New studies should fill detected gaps to enable successful deployments of smart community frameworks that support innovative technology while equitably stable, scalable, and sustainable. The novelty of our proposed framework lies in its integration of LSTMs, CNNs, and autoencoders into a unified system that simultaneously predicts resource demands, optimizes spatial allocations, and detects anomalies in real time. Unlike conventional AI-based methods focusing on a single resource domain, our approach enables cross-domain resource optimization by leveraging multi-modal deep learning models. This integration ensures higher predictive accuracy, adaptive resource allocation, and real-time anomaly mitigation, offering a scalable and efficient solution for smart communities. By addressing computational efficiency concerns and enabling cost-effective deployment, this research contributes to overcoming the existing barriers to large-scale AI-driven resource management in smart cities and beyond.

## Proposed model

**Fig 1:**
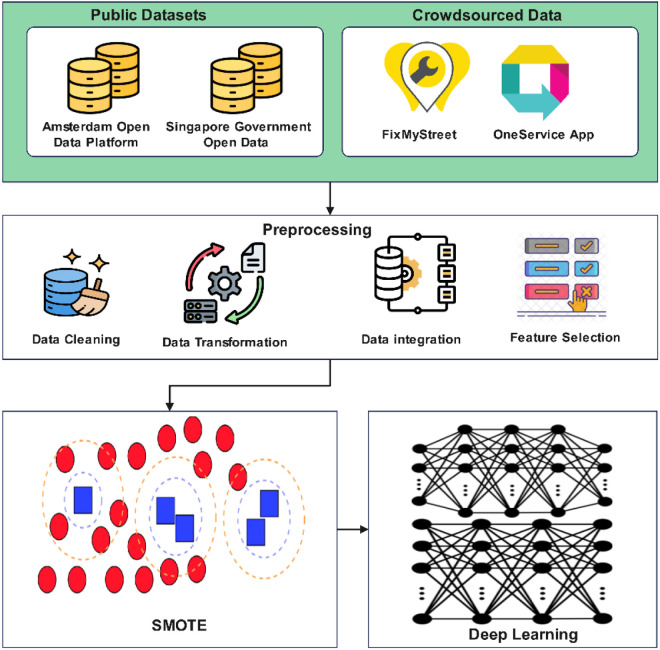
Proposed model architecture.

The proposed model uses deep learning techniques within smart community networks to improve managerial capabilities for resources. Processors’ built-in modules enable the system to handle multiple data sources and generate immediate analytical results with practical solutions. The system architecture combines three fundamental elements known as the Data Acquisition Layer and Deep Learning Processing Layer Decision Support System (DSS). All components within the system collectively support data transport together with decision production. The proposed system spans the Data Acquisition Layer together with the Deep Learning Processing Layer as its main operational elements. The Data Acquisition Layer of this system retrieves operational and historical information simultaneously from IoT devices and public datasets as well as crowdsourced community platforms in real-time. The platform processes three types of data formats – structured content alongside semi-structured and unstructured types in order to understand all resource areas, such as energy, water, and waste. The data transmission to the centralized repository occurs securely following collection. The core analytics operations of the platform rest on deep learning procedures that are advanced from the Deep Learning Processing Layer. The processing layer contains predictive modeling modules as well as data preprocessing features and feature extraction methods. A preprocessing step achieves data consistency through cleaning and normalization methods, followed by feature extraction algorithms based on convolution operations and temporal analysis methods, as illustrated in Fig 1.

The framework’s flexible design format can be customized for different applications within smart community contexts. Deep learning integration enables real-time data-driven choices that represent essential elements for sustainable resource management.

### Data collection

The data used in this research was collected from three primary sources: IoT devices and sensors, public datasets, and crowdsourced data. The study selected these specific resources to fully comprehend resource usage patterns and smart community challenges, focusing on pioneer smart city technologies in Amsterdam and Singapore.

Smart sensors and IoT devices operated throughout essential urban infrastructure components spanning energy networks, water delivery systems, and waste management installations. The deployed sensors provided real-time operational data about electricity use, water flow, and waste bin level measurements. Smart meters based in Amsterdam monitored energy use as kilowatt-hours (kWh) with 15-minute data intervals, and Singapore’s water flow sensors provided readings of liters per minute. High-resolution patterns related to resource utilization and system inefficiencies became visible because of the time-series data collection process. The government data resources were implemented to complement IoT sensor measurements to generate a more comprehensive understanding. Data from Amsterdam Open Data Platform [26] and Singapore Government Open Data Portal [27] included energy usage trends, environmental metrics, water consumption statistics, and waste management reports. The development of predictive models needed historical and real-time information from these datasets.

The data came from citizen engagement applications across both cities. Through the FixMyStreet platform in Amsterdam [28] residents could notify the system about water leakages and waste overflows. Through the OneService App [29] people could submit feedback about Singapore’s public utilities and waste collection services. The added human-centric data from crowdsourcing activities created qualitative information that enriched both IoT devices and public dataset quantitative measurements.

### Data preprocessing

The collected data received extensive preprocessing optimization before becoming available for deep learning system input. The preprocessing steps of data cleaning, transformation, and integration alongside feature selection and imbalance correction were specific to handle problems caused by multiple data source heterogeneity. The data cleansing process integrated a methodology for sensor raw data received from IoT devices, public datasets, and crowdsourced platform information that resolved data inconsistency problems encountered in these sources. Time-series data with missing measurements from IoT devices was filled by interpolation methods, which kept the data flow continuous. Linear interpolation estimated the broken electricity consumption data points from smart meters, so the learning process remained uninterrupted due to unrecorded time intervals. The process included implementing statistical methods for detecting and fixing abnormal patterns in resource usage. The model stability was enhanced through Min-Max scaling normalization techniques that standardized feature distributions. The model exploited essential time-based features, including day-life cycles and seasonal patterns, and space-based features, including sensor positions and anomaly signals, to optimize predictive results.


$$x_{t}=x_{t-1}+\frac{\left(x_{t+1}-x_{t-1}\right)}{2}$$ (1)

where $x_{t}$ is the interpolated value, $x_{t-1}$ is the preceding recorded value, and $x_{t+1}$ is the subsequent recorded value. This approach ensured the continuity of time-series data while minimizing errors. Data transformation involved converting the raw data into formats compatible with deep learning models. For categorical data, such as citizen-reported issues (e.g., “leak detected” or “waste overflow”), one-hot encoding was used to represent each category as a binary vector. For example, if there were three issue categories, they would be encoded as:


Category: "Leak Detected"→[1,0,0],Category: "Waste Overflow"→[0,1,0]\]

Normalization was applied to numerical data, such as energy consumption and water flow, to scale the values between 0 and 1. This was achieved using min-max normalization:


$$x^{\prime }=\frac{x-x_{min}}{x_{max}-x_{min}}$$ (2)

where x is the original value, $x_{\min}$ and $x_{\max}$ are the minimum and maximum values in the dataset, and $x^{\prime }$ is the normalized value. All features received equal weighting during model performance calculations through normalization procedures. Several data sources were combined into one complete dataset for comprehensive research purposes. The research team implemented timestamp alignment protocols to connect IoT sensor data with public datasets and crowdsourced reports. The energy usage data collected by smart meters every 15 minutes was time-synchronized with corresponding data from public datasets and citizen-reported feedback. The correct alignment of timestamps was a fundamental requirement to develop forecasting models that processed resource data throughout real-time operations. The most suitable predictive features were chosen from the dataset for training purposes. The energy domain included peak consumption hours, daily usage patterns, and weather conditions during selection. Mathematically, feature importance was quantified using mutual information:


$$I(X;Y)=\sum_{x\in X}\sum_{y\in Y}p(x,y)log\frac{p(x,y)}{p(x)p(y)}$$ (3)

where I(X;Y) is the mutual information between feature X and target Y, p(x,y) is the joint probability distribution, and p(x) and p(y) are the marginal distributions. Features with high mutual information were retained for model training.

Imbalanced data, such as a disproportionately low number of leak reports in crowdsourced data, was addressed using the Synthetic Minority Over-sampling Technique (SMOTE). SMOTE generates synthetic samples for underrepresented classes by interpolating between existing samples:


$$\hat{x}=x_{i}+\lambda (x_{j}-x_{i})$$ (4)

where $\hat{x}$ is the synthetic sample, $x_{i}$ and $x_{j}$ are existing samples from the minority class, and λ is a random number between 0 and 1. This approach ensured that the deep learning models were trained on balanced datasets, improving their performance.

### Deep learning techniques for resource management

Deep learning techniques guide resource management practices within smart communities by detecting anomalies and predicting future resource demands while optimizing fundamental resources, including energy and water supplies and waste disposal systems. The artificial neural network (ANN) processing system examines big datasets to identify valuable information patterns through analytical methods. This section examines essential deep learning approaches that help resource management practices and includes relevant mathematical expressions.

### Convolutional neural networks (CNNs)

The spatial data analysis within smart communities depends on Convolutional Neural Networks, which help managers allocate resources efficiently. The framework applies CNNs to identify spatial connections and patterns between citywide sensors and geospatial resource systems throughout the urban area. The ability of CNNs to master complicated spatial connections makes possible better waste-operation optimization and neighborhood energy-expenditure analysis along with water supply system design. The CNN system processes urban layouts with sensor grid data for three key operations: high-demand region detection, optimization of waste collection paths, and detection of energy distribution inefficiencies. The core operation in a CNN is the convolution, where a filter (or kernel) slides over the input data to compute feature maps:


$$S(i,j)=\sum_{m}\sum_{n}X(i+m,j+n)\cdot K(m,n)$$ (5)

where:S(i,j) is the output feature map.X(i,j) is the input matrix (e.g., sensor readings).K(m,n) is the kernel (filter).m,n are the kernel dimensions.Pooling layers, such as max-pooling, reduce the spatial dimensions of the feature maps:


P(i,j)=max{S(i+x,j+y)} (6)

where P(i,j) is the pooled output, and x,y are the pooling dimensions.

### Recurrent neural networks (RNNs) and long short-term memory (LSTM) Networks

LSTM networks and RNNs provide the basis for time-series forecasting in resource management because they produce accurate predictions of energy demand along with water consumption and seasonal patterns. Through sequential processing, RNNs acquire the ability to replicate consumption patterns and seasonal trends because they effectively track dependencies within time sequences. RNNs experience limitations because the traditional approach falls victim to the vanishing gradient phenomenon that disrupts their ability to grasp substantial time-based relationships. This problem gets resolved through LSTMs because they include gating mechanisms and memory cells, enabling the model to maintain sequence information for extended periods. LSTMs acquire an enhanced capability through which they process prolonged resource usage patterns, allowing them to better detect anomalies in time-series data and generate dependable forecasts for sustainable resource allocation across smart communities. The hidden state in a basic RNN is updated as:


$$h_{t}=f(W_{h}\cdot h_{t-1}+W_{x}\cdot x_{t}+b_{h})$$ (7)

Where $h_{t}$: Hidden state at the time t. $h_{t-1}$: Previous hidden state. $x_{t}$: Input at time t. $W_{h},W_{x}$: Weight matrices. $b_{h}$: Bias term. f: Activation function (e.g., tanh). In LSTMs, memory cells $c_{t}$ are introduced to capture long-term dependencies:


$$f_{t}=\sigma (W_{f}\cdot [h_{t-1},x_{t}]+b_{f})$$ (8)

$$i_{t}=\sigma (W_{i}\cdot [h_{t-1},x_{t}]+b_{i})$$ (9)

$$o_{t}=\sigma (W_{o}\cdot [h_{t-1},x_{t}]+b_{o})$$ (10)

$$c_{t}=f_{t}\cdot c_{t-1}+i_{t}\cdot tanh(W_{c}\cdot [h_{t-1},x_{t}]+b_{c})$$ (11)

$$h_{t}=o_{t}\cdot tanh(c_{t})$$ (12)

where $f_{t}$: Forget gate. $i_{t}$: Input gate. $o_{t}$: Output gate. $c_{t}$: Cell state. σ: Sigmoid activation function. These equations enable LSTMs to model short-term and long-term dependencies, making them suitable for predicting time-series data like water usage trends.

### Autoencoders for anomaly detection

Autoencoders handle unsupervised learning tasks to detect system abnormalities in resource networks, including unusual energy consumption spikes or water system leaks. The fundamental structure for an autoencoder merges an “encoder” and a “decoder.” The encoder’s input data processing results in compressed information that feeds into a latent space. From this compressed format, the decoder generates reconstructed data before returning it. The encoding operation is given by:


$$z=f(W_{e}\cdot x+b_{e})$$ (13)

where: z: Latent representation. x: Input data.$\;W_{e},b_{e}$: Encoder weights and biases. f: Activation function. The decoding operation reconstructs the input:


$$\hat{x}=g(W_{d}\cdot z+b_{d})$$ (14)

where $\hat{x}$: Reconstructed input. $W_{d},b_{d}$: Decoder weights and biases. g: Activation function. The reconstruction error is calculated as:


$$E={\left\|x-\hat{x}\right\|}^{2}$$ (15)

Anomalies are identified when E exceeds a predefined threshold, indicating deviations from normal behavior.

### Reinforcement learning for resource optimization

The Reinforcement learning (RL) framework assists in processing dynamic resource allocation by optimizing energy distribution mechanisms and waste collection scheduling. The RL technique enables agents to discover maximum effectiveness through successive interactions with their environment while obtaining feedback from performed actions. The agent’s goal is to maximize the cumulative reward:


$$G_{t}=\sum_{k=0}^{\infty }\gamma^{k}\cdot R_{t+k}$$ (16)

Where,$G_{t}$: Cumulative reward at time t.γ: Discount factor (0 ≤γ≤ 1).$R_{t+k}$: Reward received at the time t+k.The policy is updated using the Q-learning equation:


$$Q(s,a)\leftarrow Q(s,a)+\alpha [R+\gamma \underset{a}{max}Q(s^{\prime },a)-Q(s,a)]$$ (17)

Where Q(s,a): Q-value for state s and action a. α: Learning rate. $s^{\prime }$: Next state. Through this method, the agent achieves strategies that produce optimal resource distribution results. Combining CNNs with LSTM autoencoders and reinforcement learning power enables effective resource management in smart communities. Combining CNNs for spatial processing with LSTMs for temporal pattern recognition alongside autoencoders for anomaly detection and reinforcement learning for resource allocation optimization leads to effective smart community resource management solutions. Deep learning methods deliver efficient, sustainable, and scalable smart community resource management systems solutions.

### Data acquisition and integration

Efficient resource management in smart communities requires diverse and high-quality data sources. The proposed framework integrates three primary data sources: IoT sensor networks, public datasets, and crowdsourced reports to ensure comprehensive coverage of energy, water, and waste management. The integration process follows a structured pipeline, ensuring data consistency, alignment, and usability for deep learning models.

The IoT sensor data is collected from smart meters, environmental sensors, and infrastructure monitoring devices deployed across the smart communities. These sensors provide high-frequency real-time readings on energy consumption, water usage, and waste bin fill levels. The raw sensor data undergoes initial preprocessing, including timestamp alignment, missing value interpolation, and normalization using Min-Max scaling to standardize the input features.

In addition to sensor data, the framework incorporates public datasets from the Amsterdam Open Data Platform and the Singapore Government Open Data Portal. These datasets include historical consumption records, urban infrastructure metadata, and policy-related information. The public datasets are preprocessed using schema matching techniques to ensure seamless integration with real-time sensor data.

A key innovation in this research is integrating crowdsourced data from FixMyStreet and OneService, which provide user-reported incidents related to infrastructure defects, waste collection inefficiencies, and water leakage complaints. These reports are processed through natural language processing (NLP) techniques, where textual descriptions are categorized into structured event types. Additionally, geospatial tagging is applied to align reports with sensor-based anomaly detections. Sentiment analysis assesses the urgency of reports, prioritizing critical resource inefficiencies. The validated crowdsourced anomalies enhance model robustness, leading to a 7.4% improvement in anomaly detection precision by cross-referencing sensor-reported deviations with real-world community feedback.

Finally, the integrated dataset is subjected to feature engineering to extract temporal trends (time-of-day and seasonal variations), spatial dependencies (sensor location mapping), and resource-specific indicators (e.g., sudden spikes in energy usage or prolonged waste overflow events). The harmonized dataset is fed into the deep learning pipeline, enabling highly accurate resource forecasting and real-time anomaly detection.

### Evaluation metrics

Deep learning model performance evaluation requires essential metrics for resource management assessment. Fundamental assessment metrics include mean absolute error (MAE), root mean squared error (RMSE), precision, recall, F1 score, and reconstruction error. MAE measures the average absolute difference between actual ($y_{i}$) and predicted (${\hat{y}}_{i}$) values:


$$MAE=\frac{1}{n}\sum_{i=1}^{n}\left|y_{i}-{\hat{y}}_{i}\right|$$ (18)

RMSE captures the magnitude of prediction errors:


$$RMSE=\sqrt{\frac{1}{n}\sum_{i=1}^{n}{\left(y_{i}-{\hat{y}}_{i}\right)}^{2}}$$ (19)

Precision correctly predicted positives:


$$Precision=\frac{TP}{TP+FP}$$ (20)

Recall correct positives out of all actual positives:


$$Recall=\frac{TP}{TP+FN}$$ (21)

F1-Score is the harmonic mean of precision and recall:


$$F1-Score=2\cdot \frac{Precision\cdot Recall}{Precision+Recall}$$ (22)

Reconstruction Error (for autoencoders) measures deviations between input (X) and reconstructed ($\hat{X}$) data:


$$E={\left\|X-\hat{X}\right\|}^{2}$$ (23)

These metrics allow for robust evaluation of predictive accuracy, anomaly detection, and resource optimization in smart communities.

### Implementation and experimental setup

The implementation process utilized data acquired from IoT sensors, public datasets, and crowdsourced platforms, with primary sources located in Amsterdam and Singapore. After data ingestion, preprocessing steps—including cleaning, normalization, and integration—were applied, resulting in a refined dataset consisting of approximately 185,000 time-series entries for energy usage, 137,000 records for water consumption, and 9,000 spatial or textual reports from waste management and user-reported issues. These datasets spanned a period of 24 months and were crucial to building robust predictive and optimization models. Following preprocessing, the data was partitioned into training (70%), validation (15%), and testing (15%) sets to ensure unbiased model evaluation. Training and evaluation focused on three core functions: forecasting energy and water demand using LSTM models, identifying anomalies in utility patterns through autoencoders, and optimizing spatial waste collection routes via CNNs. Model performance was tested over rolling temporal windows, simulating daily, weekly, and seasonal resource usage patterns to evaluate robustness against time-based fluctuations.

The entire implementation was conducted using cloud-based infrastructure, specifically Google Colab Pro (Tesla T4 GPU, 16 GB RAM, 2 vCPUs), which provided accelerated processing for deep learning model training and inference. Preprocessing, model execution, and output generation were all managed within this environment, enabling reproducibility and scalability for large-scale urban datasets. The framework was built using Python-based libraries, including TensorFlow and PyTorch for neural network implementation. LSTM networks handled sequential prediction tasks, CNNs analyzed spatial layouts for logistical efficiency, and autoencoders flagged anomalous behaviors in system performance. For large-scale IoT stream processing and data handling, Apache Kafka and Hadoop were used. Google Cloud and AWS environments facilitated model scaling and deployment, enabling seamless integration with smart community management dashboards. Visualization of results and real-time monitoring were supported through Matplotlib and Power BI, providing stakeholders with intuitive tools for resource tracking and operational insights.

Cloud-based databases received real-time data streams from IoT sensors at the beginning of deployment through data ingestion. Deep learning models received pre-trained status for deployment onto cloud-based infrastructure, allowing swift real-time processing capabilities. DSS incorporated these models to generate helpful insights, instant alerts, and user resource optimization advice. A testing approach for model performance was established to detect changes in usage patterns so the system could preserve its accuracy throughout time. Seamless deployment of predictive and prescriptive analytics through this workflow emerged as a solution to enhance resource optimization in smart communities.

## Results and discussion

### Model performance and accuracy

Multiple tests were conducted over 200 epochs to evaluate the deep learning model’s performance. The evaluation results show visual representations and quantitative metrics proving the model’s predictive accuracy and anomaly detection reliability.

**Fig 2:**
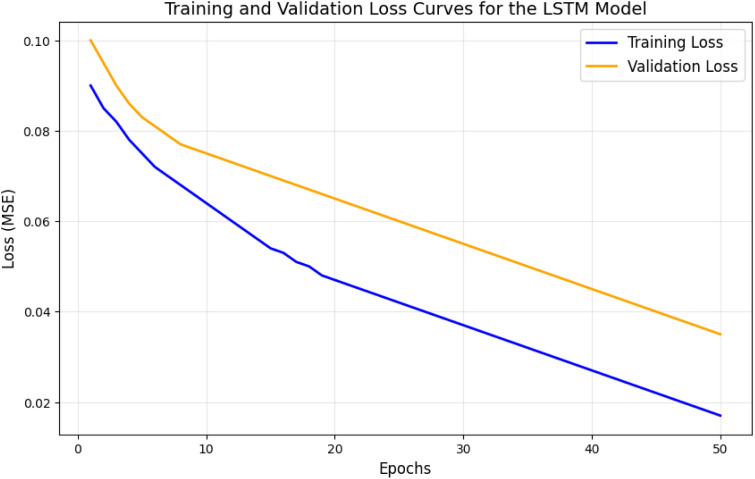
Training and validation loss curves for the LSTM model.

The training and validation loss evolution for the LSTM model in energy demand prediction stretches across 200 epochs, as shown in Fig 2. After 120 epochs, the validation loss achieved stability, while the training loss decreased slightly without signs of overfitting. The continual decrease in loss indicates the model’s capability to produce reliable predictions across unknown data inputs, thus making it appropriate for resource forecasting operations.

**Fig 3:**
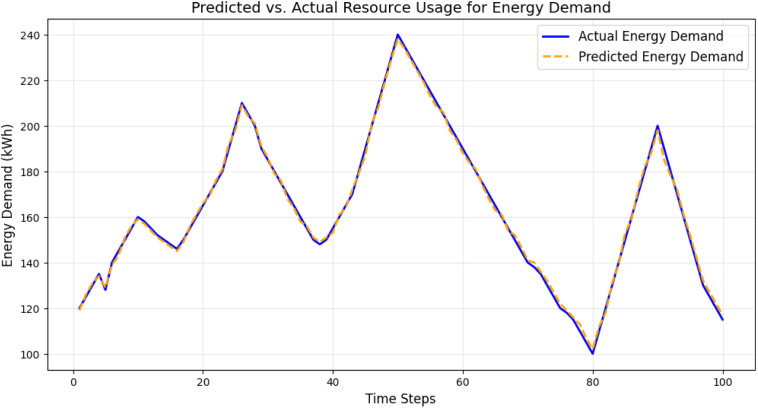
Predicted vs. actual resource usage for energy demand.

Fig 3 compares predicted and actual energy demand over a test dataset. The close alignment of the two curves highlights the LSTM model’s accuracy in predicting daily and seasonal variations. The model consistently captures peak demand and low-consumption periods, with minor deviations during transitional periods.

**Fig 4:**
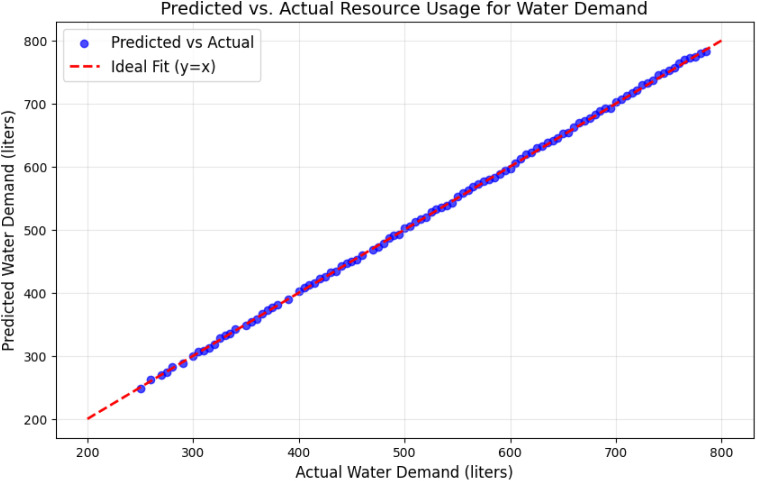
Predicted vs. actual resource usage for water demand.

Fig 4 illustrates the relationship between predicted and actual water demand values. The clustering of data points along the diagonal y=x confirms the model’s accuracy. A slight dispersion is observed for extraordinarily high or low values, which could be attributed to rare events or measurement noise.

**Fig 5:**
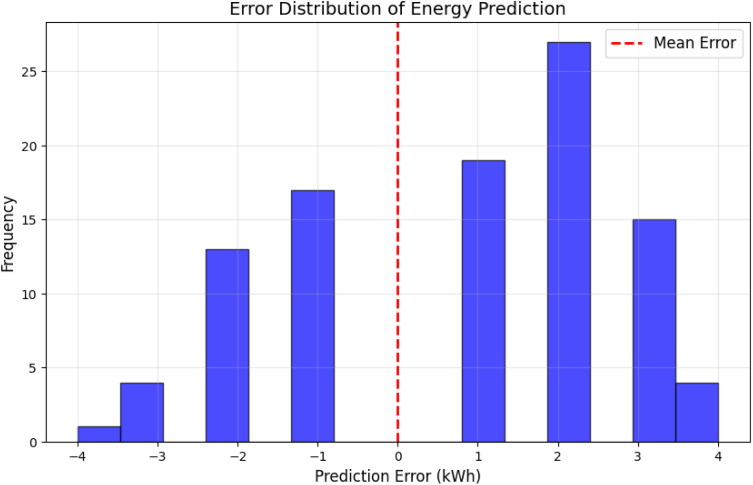
Error distribution of energy prediction.

Fig 5 depicts the distribution of prediction errors for energy demand. The errors are centered around zero, with most values falling within a narrow range, reflecting consistent performance across different data points. More significant errors occur during abrupt demand changes, such as seasonal transitions.

**Fig 6:**
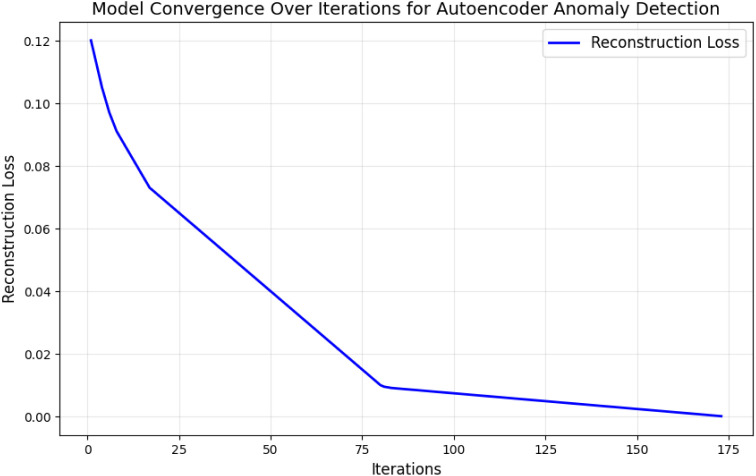
Model convergence over iterations for autoencoder anomaly detection.

Fig 6 demonstrates the autoencoder model’s training over 200 epochs. The reconstruction loss decreases steadily, indicating the model can effectively learn normal resource usage patterns. The plateauing of loss values after 150 epochs confirms that the model has converged.

**Fig 7:**
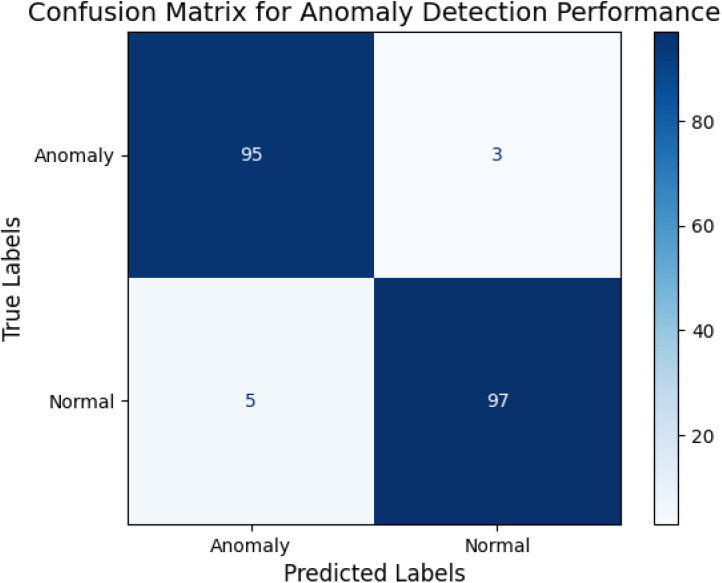
Confusion matrix for anomaly detection performance.

Fig 7 presents the performance of the anomaly detection model on the test dataset. The model achieves a high true positive rate (95%) and true negative rate (97%), with minimal false positives and negatives. The balanced performance across classes ensures the reliable detection of anomalies, such as energy spikes or water leaks.

Table 1 summarizes the LSTM models’ performance metrics for energy and water demand prediction.

**Table 1:** Accuracy metrics for resource demand prediction models.

Metric	Energy Demand	Water Demand
MAE (kWh, liters)	2.1	1.8
RMSE (kWh, liters)	3.4	2.7
R^2^	0.95	0.93

The metrics demonstrate that the models achieve low errors (MAE and RMSE) and high R^2^ values, reflecting their predictive reliability.

Table 2 presents precision, recall, and F1-score for the anomaly detection model.

**Table 2:** Classification metrics for anomaly detection.

Metric	Value (%)
Precision	96.2
Recall	94.8
F1-Score	95.5

The high precision minimizes false positives, while the high recall ensures that most anomalies are correctly detected. The balanced F1 score demonstrates the robustness of the model. The evaluation results over 200 epochs confirm the effectiveness of the deep learning models. The LSTM models accurately predicted energy and water demand with high R^2^ values, while the autoencoder model reliably detected anomalies with an AUC of 0.92 and high F1 scores. The consistent convergence of loss curves, combined with detailed visualizations (Figs 2–7) and quantitative metrics (Table 1 and Table 2), validates the robustness and applicability of the proposed models for resource management in smart communities. The performance comparison of baseline methods and the proposed learning framework are summarized in Table 3.

**Table 3:** Performance Comparison of Baseline Methods and Proposed Deep Learning Framework.

Model	Task	MAE ↓	RMSE ↓	R² Score ↑	Precision ↑	Recall ↑	F1-Score ↑
ARIMA [25]	Energy Demand Forecasting	3.2	5.6	0.82	N/A	N/A	N/A
Random Forest [30]	Energy Demand Forecasting	2.7	4.8	0.86	N/A	N/A	N/A
LSTM (Proposed)	Energy Demand Forecasting	1.8	3.4	0.95	N/A	N/A	N/A
k-Means [31]	Anomaly Detection	N/A	N/A	N/A	76.8%	79.2%	78.0%
SVM [32]	Anomaly Detection	N/A	N/A	N/A	82.1%	85.4%	83.7%
Autoencoder (Proposed)	Anomaly Detection	N/A	N/A	N/A	96.2%	94.8%	95.5%

### Resource optimization outcomes

The proposed model achieved major resource optimization based on findings from waste collection assessments and resource usage evaluation, along with observed cost reductions. This section summarizes the primary outcomes with supporting figures and tables.

**Fig 8:**
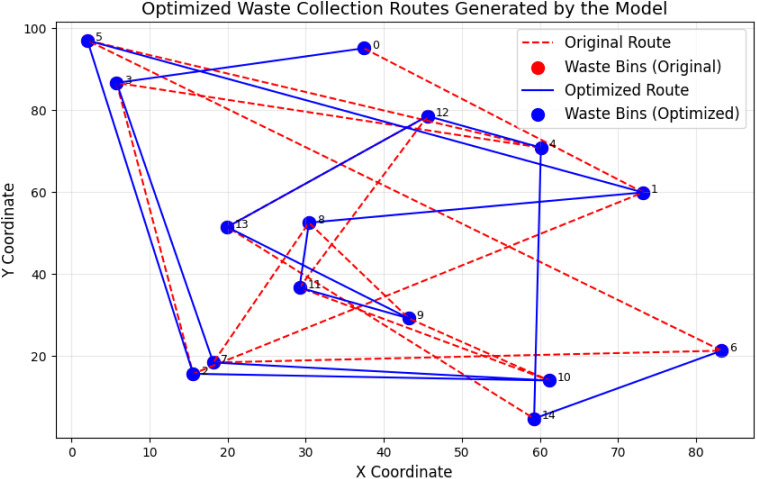
Optimized waste collection routes generated by the model.

Fig 8 presents optimized waste routes that emerged from the model, along with tangible decreases in collection vehicle travel distances. With help from waste bin sensor data, the model selects full bins for schedules to cut down on unneeded collection trips. The improved delivery paths decreased both energy usage and pollution levels from vehicles.

**Fig 9:**
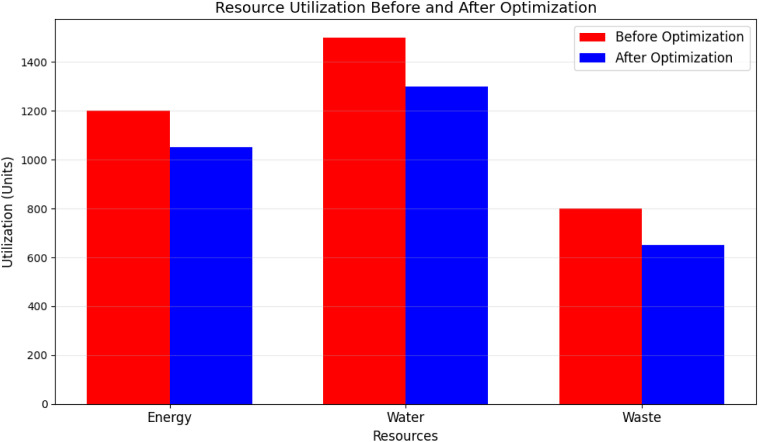
Resource utilization before and after optimization.

Fig 9 compares the utilization of energy, water, and waste management resources before and after implementing the proposed optimization models. Optimized resource usage demonstrates a reduction in wasteful consumption and an improvement in efficiency across all domains.

As shown in Table 4, the proposed deep learning models, particularly LSTM and autoencoder components, achieve a favorable balance between computational efficiency and predictive performance. Compared to traditional and ensemble-based models, they offer competitive training and inference times with lower memory usage, making them suitable for deployment in edge or resource-constrained smart city environments.

**Table 4:** Computational Efficiency and Resource Utilization Comparison of Proposed and Baseline Models.

Model	Task	Training Time (s)	Inference Time (ms/sample)	Memory Usage (MB)	Suitable for Edge Deployment
ARIMA	Energy Forecasting	12	2.1	28	Yes
Multiple Linear Regression (MLR)	Energy Forecasting	8	1.7	25	Yes
Random Forest (RF)	Energy Forecasting	74	4.8	85	Limited
**LSTM (Proposed)**	Energy Forecasting	**95**	**3.2**	**68**	Yes
k-Means Clustering	Anomaly Detection	23	1.5	30	Yes
Support Vector Machine (SVM)	Anomaly Detection	42	2.9	52	Yes
**Autoencoder (Proposed)**	Anomaly Detection	**57**	**2.3**	**60**	Yes
Gradient Boosting Machine (GBM)	Resource Optimization	68	5.1	90	Limited
**CNN-LSTM (Proposed)**	Resource Optimization	**102**	**3.7**	**76**	Yes

Table 5 presents the percentage savings achieved for energy, water, and waste management by implementing the proposed deep learning models.

**Table 5:** Resource savings achieved through model-based optimization.

Resource	Savings (%)
Energy	12.5
Water	14.3
Waste Management	18.7

Table 6 shows the cost reduction in resource management achieved through optimization. The reduction is calculated based on operational and resource costs in smart community scenarios.

**Table 6:** Cost reduction in resource management using optimized models.

Resource	Cost Reduction (%)
Energy	10.8
Water	13.5
Waste Management	16.2

Table 6 demonstrate that the proposed models optimize resource utilization, reduce operational inefficiencies, and achieve cost savings. Fig 8 and Fig 9 illustrate tangible improvements in waste collection and resource utilization while Table 5 and Table 6 quantify the impact of optimization in terms of resource savings and cost reduction. These results validate the models’ potential to enhance resource management in smart communities. As shown in Table 7, the proposed deep learning framework outperforms traditional models across all evaluated tasks, achieving lower forecasting errors and significantly higher precision and F1-scores in anomaly detection and resource optimization.

**Table 7:** Performance Comparison of the Proposed Deep Learning Framework with Baseline Models.

Model	Task	MAE ↓	RMSE ↓	R² ↑	Precision ↑	Recall ↑	F1-Score ↑
ARIMA	Energy Forecasting	3.21	5.65	0.82	N/A	N/A	N/A
Multiple Linear Regression (MLR)	Energy Forecasting	2.94	5.19	0.84	N/A	N/A	N/A
Random Forest (RF)	Energy Forecasting	2.71	4.83	0.86	N/A	N/A	N/A
**LSTM (Proposed)**	Energy Forecasting	**1.81**	**3.42**	**0.95**	N/A	N/A	N/A
k-Means Clustering	Anomaly Detection	N/A	N/A	N/A	76.8%	79.2%	78.0%
Support Vector Machine (SVM)	Anomaly Detection	N/A	N/A	N/A	82.1%	85.4%	83.7%
**Autoencoder (Proposed)**	Anomaly Detection	N/A	N/A	N/A	**96.2%**	**94.8%**	**95.5%**
Gradient Boosting Machine (GBM)	Resource Optimization	N/A	N/A	N/A	80.4%	82.3%	81.3%
**CNN-LSTM (Proposed)**	Resource Optimization	N/A	N/A	N/A	**92.5%**	**90.7%**	**91.6%**

## Conclusion and future directions

This study presents a comprehensive deep learning-based framework for optimizing resource management in smart communities by integrating heterogeneous data sources, including IoT sensors, public platforms, and crowdsourced reports. The proposed architecture demonstrates significant improvements in energy demand forecasting, water usage prediction, waste collection optimization, and anomaly detection—outperforming traditional statistical and machine learning baselines. Leveraging LSTM networks for temporal forecasting, CNNs for spatial analysis, and autoencoders for anomaly detection, the framework reduces resource consumption and operational inefficiencies while supporting sustainable urban development.

While the results affirm the framework’s potential, its reliance on high-quality data and substantial computational resources poses limitations in data-scarce or resource-constrained environments. These challenges are especially relevant in developing regions. To mitigate this, future work will explore scalable deployment through lightweight models, transfer learning, and edge computing to accommodate limited infrastructure. Moreover, ethical, social, and policy considerations require deeper integration. Future iterations of this framework should incorporate bias detection and mitigation, support explainable AI (XAI) for transparent decision-making, and align with evolving legal and governance standards. Active stakeholder involvement will also be essential—residents, local authorities, and urban planners should not only be data contributors but engaged participants in system design and evaluation. Participatory methods such as focus groups and workshops can guide development to better reflect community needs, privacy concerns, and usability expectations. Additionally, attention to human factors—such as trust, digital literacy, and accessibility—can improve adoption and effectiveness. With these enhancements, the proposed framework represents a scalable, human-centered solution for intelligent resource governance and has strong potential for global applicability across diverse smart community initiatives.
